# The Achievements of Science

**Published:** 1861-06

**Authors:** 


### THE ACHIEVEMENTS OF SCIENCE.

NEVER, in the history of the world, has science been more actively and efficiently engaged in pushing its researches, than now; and mainly because this is an age of peace. Hitherto war has been the rule—peace the exception. Now, it is the reverse. Time is allowed to men to apply their mental energies to more elevated and useful purposes than slaying one another, pillaging cities, and subverting empires. The steam-engine saves labor; the telegraph economizes time; hence, less work, greater comfort, and more leisure are secured to the busy brain-worker — leisure for devising appliances which shall be the instrumentalities of a higher civilization, at once ennobling and happifying. Horrid wars, in the past, destroyed the populations; gentle peace, in the present, increases them. But to preserve the increasing millions physically science must be appealed to; morally, religion. Thus it is that, in every year of the world's future history, science will become more perfectly the hand-maid of religion, and they will be co-workers in making this earth an Arcadia more enrapturing than any of which philosopher ever dreamt, or poet sang, but which the prophets of Divinity pre-shadowed in the declaration: "The desert shall bud and blossom as the rose." A double verification; for while science will cover the Saharas of the world with waving grass and bending corn, our holy religion will fructify the moral wastes and make of earth a paradise fit for the home of angels.

In proportion as the population of the world increases, the aids of science are becoming more and more indispensable towards making two blades of grass grow where before there grew but one; and the acre of to-morrow must yield the double of to-day's. Hence, a brighter and a better day is dawning for men of mind—for those who possess inventive genius and combine with it the industry and the love of its exercise and application. Hard is the heart which does not sorrow over the ill requital of the men of a generation or two agone, whose whole lives were expended in wearing anxiety of mind and wasting toil of body, in poverty, if not even in destitution, in eliminating machineries which were destined to enrich those whom they never knew; in whose veins no kindred blood flowed, while they themselves were to end their labors and their lives in sight of fruitions which the hands of them and theirs were never to gather!

It was a sad record of two weeks ago (*Scientific American*, page 276) that, in a single branch of an industrial department, the men who, during the last century, initiated machineries which now fill the mouths of millions of the two greatest nations on earth with bread, died miserably poor; and some of their immediate descendants were only saved from death by want, through public pity! The prospect, however, is cheering, that a better fate and a higher reward await the Kays, and

Pauls, and Higbeys, and Hargreaves, and Whitneys, of the present and coming generations, and that they will become the Arkwrights, the Cramptons, and the Peels of our own time, for because of them "Cotton is King!"

Whatever may have been the demands of past ages, inventive genius is the necessity of the present. If the sword has hitherto reigned supreme, science must be its successor. The sword may initiate or construct an empire, but science, in its application to industrial pursuits, in the direction of machineries for manufactories, and implements for farms, must be invoked to sustain it. Nations can live by the sword no longer, for the dominion of barbarism has passed away, and empire must be humanitarian and Christian, founded on true knowledge and its wise application.

